# Co-morbidities of persons dying of Parkinson's disease

**DOI:** 10.1179/1743291X12Y.0000000037

**Published:** 2013-07

**Authors:** Lynn Lethbridge, Grace M Johnston, George Turnbull

**Affiliations:** 1 School of Health Administration, Faculty of Health Professions, Dalhousie University, Halifax, Nova Scotia, Canada; 2 School of Health Administration, Faculty of Health Professions, Dalhousie University, Halifax, Nova Scotia, Canada; and Surveillance and Epidemiology Unit, Cancer Care Nova Scotia, Halifax, Nova Scotia, Canada; 3 School of Physiotherapy, Faculty of Health Professions, Dalhousie University, Halifax, Nova Scotia, Canada

**Keywords:** Parkinson disease, Palliative care, Advance care planning, Death certificates, Co-morbidity

## Abstract

**Introduction:**

Disease interactions can alter functional decline near the end of life (EOL). Parkinson's disease (PD) is characterized by frequent occurrences of co-morbidities but data challenges have limited studies investigating co-morbidities across a broad range of diseases. The goal of this study was to describe disease associations with PD.

**Methods:**

We conducted an analysis of death certificate data from 1998 to 2005 in Nova Scotia. All death causes were utilized to select individuals dying of PD and compare with the general population and an age–sex-matched sample without PD. We calculated the mean number of death causes and frequency of disease co-occurrence. To account for the chance occurrence of co-morbidities and measure the strength of association, observed to expected ratios were calculated.

**Results:**

PD decedents had a higher mean number of death causes (3.37) than the general population (2.77) and age–sex-matched sample (2.88). Cancer was the most common cause in the population and matched sample but fifth for those with PD. Cancer was one of nine diseases that occurred less often than what would be expected by chance while four were not correlated with PD. Dementia and pneumonia occurred with PD 2.53 ([CI] 2.21–2.85) and 1.83 (CI 1.58–2.08) times more often than expected. The strength of association for both is reduced but remains statistically significant when controlling for age and sex.

**Discussion:**

Those with PD have a higher number of co-morbidities even after controlling for age and sex. Individuals dying with PD are more likely to have dementia and pneumonia, which has implications for the provision of care at EOL.

## Introduction

Parkinson's disease (PD) is a neurodegenerative disease estimated to afflict 100 000 individuals in Canada. Damaged or dying cells inhibit the production of the neurotransmitter dopamine causing various motor symptoms including tremor, bradykinesia, muscle rigidity, impaired balance, and reduced facial expression.^1^ Health service requirements are particularly acute as death approaches, as symptoms become more difficult to control with medications,^2^ and persons often become bed ridden as a result of fractured hips due to falls and pneumonia due to aspiration.^3^ The health needs of persons with PD are further complicated by the frequent occurrence of co-morbidities.^4–7^ Persons with PD also have significant associations with non-motor conditions including anxiety and depression.^8,9^

Tracking the underlying cause of death from death certificates is an important aspect of disease surveillance. However, death is often due to multiple causes resulting in difficulties when assigning a single cause. There can be disagreements and coding process problems, particularly for older adults.^10,11^ Furthermore, utilizing only the underlying cause can underestimate disease populations including for those with PD.^3,12–14^ Multiple causes of death can be used to provide a more comprehensive disease profile and thereby capture a more complete description of the PD and other disease-specific populations that could benefit from palliative care.

Understanding the strength of associations between diseases can assist in planning for palliative care for persons with PD. Research on co-morbidities among persons with PD has been limited due, in part, to data restrictions. Prospective studies have been problematic as there is no definitive test for PD^15,16^ resulting in a high rate of false positives.^17^ Disease registries are a valuable resource in epidemiological studies; however, a PD registry does not currently exist in Canada. As well, controlled trials often exclude older individuals and those with multiple co-morbidities.^18,19^ In terms of PD research, most previous co-morbidity studies have focused on a single or restricted number of conditions.^4^ Access to data on all causes of death provides an opportunity to investigate multiple disease associations at a population level. A wide range of disease associations can be examined while controlling for patient characteristics.

The overall objective of this study is to gain an understanding of the health conditions near the end of life (EOL) for those with PD. Specifically, we analyze all causes of death information to identify conditions that are associated with PD and provide a measure of the strength of that association.

## Methods

The study population included all residents of Nova Scotia, Canada who died from 1 January 1998 to 31 December 2005 (*N* = 63 431) as identified from the Nova Scotia Vital Statistics death certificate database. Since the Statistics Act in Canada requires the registration of all deaths, Vital Statistics files are virtually complete.^20^

Cause of death on the death certificate was used to identify diseases present in the study population. Up to 13 causes can be recorded. The International Classification of Diseases tenth revision (ICD-10) was used for 2000 forward, with previous years utilizing the ninth revision (ICD-9). The PD population includes decedents with any mention of PD as a cause of death defined using ICD-10 codes G20–G21 and ICD-9 code 332 which include PD and secondary Parkinsonism.

Those with PD were compared with all deaths in the province to determine whether co-morbidity patterns for the PD group differed from that of all the decedents. This examination is useful for understanding how persons with PD differ from all persons at EOL.

To adjust for age and sex differences between the PD decedents and all deaths, each PD decedent was matched to a non-PD decedent by sex and year of age. The point estimate statistics were calculated from this matched sample. Since there are a limited number of decedents with PD, a bootstrap procedure was used to measure statistical variation. Specifically, the matching procedure was repeated 200 times and the standard error was calculated as the standard deviation of the bootstrapped point estimates. The mean of the bootstrapped estimates is not used as a measure of the point estimates as this is a biased estimate of the parameter.^21^

Through focus groups, interviews, and literature, Rosenwax *et al.*^22^ developed minimal, mid-range, and maximal estimates of population groups who could potentially benefit from palliative care based on disease types. We selected 15 of the maximal conditions plus a group of causes defined as ‘injuries, poisonings and external cause of death’, as fractures have been shown to be correlated with PD.^23,24^ (Due to confidentiality restrictions on data access, we are unable to separate injuries, poisonings, and other external causes into individual categories.) The frequency of occurrence of each condition was calculated across three populations; those with PD, the general population, and the age–sex-matched sample without PD. Co-morbid disease frequencies were ranked from highest to the lowest for all three groups.

Since multiple chronic diseases affect a large percentage of the population, two or more conditions may occur jointly simply due to the high prevalence of the diseases. To control for the chance occurrence of PD with other diseases, the ratio of observed to expected deaths was calculated where PD is mentioned together with each of the 16 selected diseases groups.^10^ The strength of association is calculated as
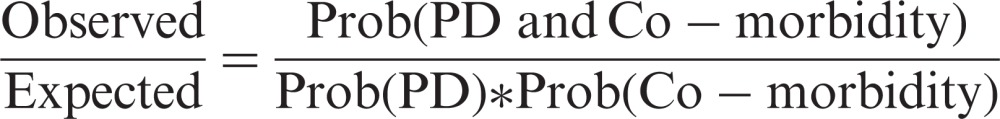


This is the proportion with both conditions (i.e. the observed) divided by the proportion with PD multiplied by the proportion with the co-morbidity. As shown by the probability theory, the product of the probabilities of two events occurring is equal to the probability of both occurring together, if the events are independent.

If the ratio of observed to expected is greater than 1, the association is greater than expected, while a value less than 1 indicates an association which is less than expected. The association between diseases was considered independent if the 95% confidence interval around the ratio included one. That is, the observed number of decedents with both diseases on a death certificate is not significantly different from what is expected by chance.

Ethics approval was granted from Capital Health Research Ethics Board. All analyses were conducted using SAS 9.2.

## Results

**Figure 1:**
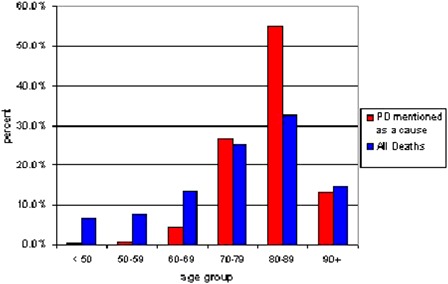
Comparison of age distribution – those with PD as a cause and all deaths.

Among the 63 431 decedents, 900 (1.4%) had PD mentioned as a cause on the death certificate. Less than half of the PD decedents, 418 (46.4%), had PD listed as the underlying cause of death. Fig. 1 shows the age distribution for those with PD mentioned compared with all decedents. Over half of those with PD (54.8%) were 80–89 years of age compared with 32.4% for all decedents. Those with PD had a mean age of 82.0 compared to all decedents averaging 74.9 years. A higher percentage of those with PD mentioned were male (52.8%) compared with all decedents (50.1%).

**Figure 2:**
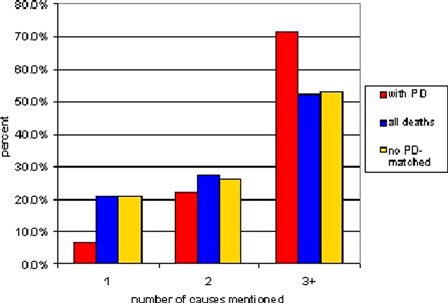
Comparison of total number of causes on death certificates.

Fig. 2 shows a comparison of total causes mentioned among those with PD, all deaths and the age–sex-matched sample without PD. About 71.5% of those with PD had three or more causes listed compared with 52.1% of all decedents. The sample without PD but having the same age and sex structure as those with PD showed a similar pattern to all deaths with 53.1% having three or more causes. The overall mean number of causes was 3.37 for those with PD, 2.77 for all deaths, and 2.88 for the matched sample.

Table 1 indicates the most frequently mentioned causes for each of the three groups. Dementia and pneumonia were mentioned most often as other causes of death for those with PD at 26.2 and 22.3%, respectively. These rates were significantly higher than the corresponding rates of 10.4 and 12.2% for all decedents. Among the age–sex-matched sample without PD, 11.7 and 14.1% mentioned dementia and pneumonia, respectively.

**Table 1:** Percentages and confidence intervals for co-morbidities mentioned on death certificate for persons dying of Parkinson's disease, all decedents, and an age–sex-matched sample, Nova Scotia, 1998–2005

Co-morbidity	Parkinson's cases		All decedents		Matched sample without PD	
	Percentages*(%)	Rank	Percentages*	Rank	Percentages*	Rank
Alzheimer's disease/dementia	26.2 ** (23.3–29.2)	1	10.4 (10.1–10.6)	8	11.7 (9.6–13.8)	9
Pneumonia	22.3** (19.6–25.1)	2	12.2 (11.9–12.4)	3	14.1 (12.0–16.2)	4
Cerebrovascular disease (stroke)	14.2 (11.9–16.6)	3	12.1 (11.9–12.4)	4	13.4 (11.1–15.7)	5
Chronic ischemic heart disease	13.4** (11.2–15.7)	4	18.9 (18.6–19.2)	2	20.7 (18.0–23.4)	2
Malignant neoplasms (cancer)	11.1** (9.0–13.2)	5	33.0 (32.6–33.4)	1	29.6 (26.7–32.4)	1
Congestive heart failure	8.1** (6.3–10.0)	6	11.3 (11.0–11.5)	6	13.2 (11.1–15.4)	6
Diabetes	8.0** (6.2–9.8)	7	10.9 (10.7–11.1)	7	12.6 (10.5–14.6)	7
Injuries, poisonings and external cause of death	7.2 (5.5–9.0)	8	8.4 (8.1–8.6)	12	7.1 (5.6–8.6)	12
Essential hypertension	6.9** (5.2–8.6)	9	8.9 (8.6–9.1)	10	11.0 (9.0–13.0)	11
Chronic obstructive pulmonary disease	6.2** (4.6–7.9)	10	11.6 (11.4–11.9)	5	14.8 (12.7–16.9)	3
Renal failure	4.2** (2.9–5.6)	11	8.8 (8.6–9.1)	11	11.1 (9.2–13.0)	10
Acute myocardial infarction	4.2** (2.9–5.6)	12	10.2 (9.9–10.4)	9	12.2 (10.5–14.0)	8
Peripheral vascular disease	3.7** (2.4–5.0)	13	6.2 (6.0–6.4)	13	6.8 (5.1–8.4)	13
Septicemia	2.4 (1.4–3.5)	14	3.3 (3.1–3.4)	14	3.7 (2.5–4.9)	14
Skin Cancer	0.22 (0.0–0.6)	15	0.40 (0.4–0.5)	15	0.22 (0.0–0.54)	15
Multiple Sclerosis	0 (0.0–0.4)	16	0.25 (0.2–0.3)	16	0.0	16
Total decedents	900		63 431		1800	

*95% Confidence interval in parentheses.

**PD percentage is statistically different from all decedents and the age–sex-matched sample.

Cancer was the most common cause of death for all decedents, occurring in 33.0% of all death certificates. Considerably fewer, 11.1%, of those with PD had cancer as a cause of death. For the age–sex-matched sample without PD, 29.6% had cancer listed.

The observed/expected ratios are given in Table 2. Dementia and PD appear together on the death certificate 2.53 times more often than expected. Similarly, pneumonia appears with PD 1.83 times more often. Nine diseases were significantly less than expected: essential hypertension, diabetes, congestive heart failure, chronic ischemic heart disease, peripheral vascular disease, chronic obstructive pulmonary disease, renal failure, acute myocardial infarction, and cancer. The ratios for stroke, injuries, septicemia, and skin cancer were not significant, indicating that these diseases occur among persons with PD at the same rates as observed in the general population. There were no instances of PD and multiple sclerosis occurring together on a death certificate.

**Table 2:** Causes of death associated with PD: comparison with all decedents and an age–sex-matched sample

Co-occurring causes of death	Ratio of observed to expected co-occurrence of cause of death	
	All decedents	Matched sample
Alzheimer's disease/dementia	2.53*	1.38*
Pneumonia	1.83*	1.23*
Cerebrovascular disease (stroke)	1.17	1.03
Injuries, poisonings and external cause of death	0.87	1.01
Essential hypertension	0.78*	0.77*
Septicemia	0.75	0.80*
Diabetes	0.73*	0.78*
Congestive heart failure	0.72*	0.76*
Chronic ischemic heart disease	0.71*	0.79*
Peripheral vascular disease	0.59*	0.70*
Skin cancer	0.56	1.00
Chronic obstructive pulmonary disease	0.54*	0.59*
Renal failure	0.48*	0.55*
Acute myocardial infarction	0.41*	0.51*
Malignant neoplasms (cancer)	0.34*	0.55*
Multiple sclerosis	–	–
observations	63 431	1800

*Statistically significant at 95% if confidence interval does not include 1.

Standard errors for matched sample calculated as the standard deviation of 200 bootstrapped estimates.

Note: – indicates no cases with both diseases listed.

Comparing the PD decedents to the age–sex-matched sample revealed similar findings. Dementia and pneumonia occurred together with PD more often than expected. The same nine diseases occur together with PD less frequently than expected while stroke, injuries, and skin cancer again were not significantly significant. The association with septicemia was not significant when compared to the full population but appeared less frequently than expected for the PD decedents compared to the age–sex-matched sample. Differences were observed in the magnitude of the associations of the PD decedents with the age–sex-matched sample and the total population. The ratios for dementia and pneumonia are smaller (1.38 and 1.23) when controlling for age and sex while the nine conditions less likely to occur with PD have ratios closer to one with the exception of essential hypertension which is nearly identical.

## Discussion

Consistent with previous research,^25–27^ results from this study indicate that those dying with PD are older and have a higher number of co-morbidities compared with the population in general. Although age and co-morbidities have been shown to be correlated,^28^ our results show those with PD have a higher number of causes of death cited even when compared with those without PD who have the same age distribution. Our findings that dementia and pneumonia are mentioned more frequently with PD than expected are consistent with previous research^3,4,23,24,29,30^ and thereby provides construct validity for this EOL care planning method. As well, our findings correspond with previous studies which have shown that persons with PD have a decreased risk of cancer.^31–33^ Genes predisposed to PD may have a protective effect against malignant tumor growth.^34,35^ Also, there is evidence that smoking, a risk factor for cancer, reduces the likelihood of PD^36–39^.

Aggregating all cancers into a single group may mask any indication of association between PD and a particular cancer type. An increased risk of skin cancer among persons with PD has been established in the literature.^32,40^ However, the study findings did not show a statistically significant association between PD and skin cancer possibly because only a relatively small number of individuals have skin cancer listed as a cause of death, leading to the potential for inadequate statistical power. Also, there is research that suggests inaccuracies involving the coding of skin cancer on death certificates are particularly problematic.^41,42^

Since all causes of death were utilized, cancer rates were higher than those measured using the underlying cause only.^43^ Note as well, statistics have shown a higher percentage of deaths due to diseases of the circulatory system compared with cancer in Canada.^43^ In this study, the circulatory diseases grouping is stratified into smaller categories so each will appear less frequently.

An association between Parkinson's and septicemia was not observed, which is inconsistent with previous studies that have shown a positive association.^24,44^ This may be due in part to the underreporting of septicemia on death certificates.^45^ Fractures are a common PD co-morbidity.^6,23,24,30,46^ However, as noted, our study data did not allow fractures to be separated from other external causes of death, limiting the statistical precision of the specific association.

The inherent challenges in primary data collection for EOL studies such as ethical issues and frailty of study subjects necessitate the use of secondary data sources, including death certificate information. Utilizing all causes of death from death certificate information enables an analysis across a broad range of conditions. Death certificates have previously been used to understand and plan for persons dying of cancer^47–49^ and are well-suited to examine similar issues for those with PD.

While difficulties in determining cause of death has led to skepticism regarding the reliability of death certificate information,^3^ the validity of cancer as a cause of death has been demonstrated.^50^ Parallel validation of PD as a cause of death can be carried out in the future by comparing vital statistics diagnoses to hospital separation and physician claims data. Linkage of multiple data sources including census data would also allow for the inclusion of additional control variables and the investigation of the observation of Pressley *et al.*^13^ that there are socio-economic differences as higher income PD patients are more likely to have PD listed as a cause of death. Furthermore, death certificate data can be used to generate Lunney *et al.*^51^ trajectories of functional decline at the EOL which have implications for optimal palliative care.

An important finding in this work is the higher than expected occurrence of dementia as a cause of death. In fact, it shows the strongest association with PD of all the study conditions, yet research has indicated that dementia is under-reported on death certificates. Tinetti *et al.*,^52^ for example, show that a higher proportion of deaths can be attributed to dementia than what appears on death certificates. A prospective study which followed nursing home residents with end-stage dementia showed 37% of cases did not list dementia anywhere on the death certificate.^53^ It has been suggested that physicians sometimes may not record dementia for various reasons such as a perceived stigma attached with the condition, reimbursement concerns, or that it is considered just a normal part of aging.^54^ Among the general population, our results show that dementia is the eighth most frequently mentioned co-morbidity and ninth for the matched sample without PD, yet for those with PD it is ranked first. This may indicate that any resistance to recording dementia may be diminished for those where PD is also listed.

Death certificate data have also been shown to underestimate the prevalence of PD.^3,12,13,55^ Therefore, all individuals with PD may not have been captured in this study. This limitation is mitigated by the utilization of all causes of death rather than focusing on the underlying cause only.^14^ Also, the large population-based sample across multiple years helps to diminish the effects of variations in death certificate coding over time and across individuals. Finally, the number of variables available to control for confounding factors is limited with the use of death certificate data in isolation of linkage to other databases which could be incorporated in future studies.

In conclusion, death certificates are a readily-available, cost-effective, population-based data source, which enables analyses across a broad range of conditions to aid in planning for care at EOL for persons with chronic diseases. The death certificate findings reported herein are consistent with the PD literature. Future work should include linkages to health services and palliative care data to further validate findings and gain a greater understanding into patterns of care near the EOL.

As the population ages, improving our understanding of the care needs of persons with advanced PD will become increasingly relevant to policy-makers and health administrators. Co-morbid conditions associated with advanced PD add to the disease burden and contribute to higher resource utilization.^13^ Monitoring and understanding disease associations with PD can assist in planning for optimal health resource allocation. The method used herein to provide evidence to help plan EOL care for persons with PD can be adapted to assist in the planning of palliative care for other chronic diseases.
